# Brain Surgery

**Published:** 1893-10

**Authors:** 


BRAIN SURGERY.—By M. Allen Starr, M. D., Ph. D., Professor of Diseases of the Mind and Nervous System, College of Physicians and Surgeons, Medical Department of Columbia College, New York; President of the New York Neurological Society; Consulting Neurologist to the Presbyterian, Orthopædic, and Babies Hospitals. With fifty-nine Illustrations. Octavo, 308 pages, extra muslin, price $3.00. New York: William Wood & Company.

This book embraces one of the most popular subjects, at this time, in the whole domain of surgery. So much attention has been given to brain surgery during the past few years, the subject is so new and the possibilities in this llne are so great that all are interested in any work which treats of it. In the preparation of the work before us Dr. Starr has demonstrated his special fitness for the task assumed, and the book will be hailed with delight everywhere. In its preparation the author has endeavored to utilize American observations and to cite American cases in preference to others. This has in no way hampered, for it is, as he says "To the industry and genius of American surgeons that much of the great advance in this department of surgery is due." Quite a number of the cases reported and of the investigations given are from the author's own work and experience.

The author discusses the subject of brain surgery under the following heads: Diagnosis of Cerebral Disease, Trephining for Epilepsy, Trephining for Imbecility due to Microcephalus, Trephining for Cerebral Hemorrhage, Trephining for Abscess of the Brain, Trephining for Tumor of the Brain, Trephining for Hydrocephalus and for the Relief of Intracranial Pressure, Trephining for Insanity, Trephining for Headache and for other conditions, the Operation of Trephining.

It will be noticed from the foregoing sub-headings that the book is devoted to diseases of the brain rather than acute injuries and wounds. A large part of the work is devoted to the consideration of Jacksonian Epilepsy and its surgical treatment. The author states, that the operation of trephining for epilepsy has been done more than 300 times during the last five years; 42 cases operated on are reported in this book, 13 of which occurred in the author's own practice. Of the 42 cases reported, 13 were cured, 11 were improved, 15 not improved, and 3 died.

A number of cases, in the hands of the best surgeons, are reported under the various sub-headings.

The chapter on the operation of trephining is especially instructive. The preparation of the patient, the selection of instruments and surgical dressings, the operation proper, the dressing of the wound and the after treatment are fully discussed. Altogether the book is a very meritorious one and will doubtless become very popular.

Whether all of the views of the author will stand the test of time and further research remains to be seen. This subject is so new and the field apparently so wide that it is difficult to even conjecture its boundaries.

